# Characterisation of a Peripheral Neuropathic Component of the Rat Monoiodoacetate Model of Osteoarthritis

**DOI:** 10.1371/journal.pone.0033730

**Published:** 2012-03-21

**Authors:** Matthew Thakur, Wahida Rahman, Carl Hobbs, Anthony H. Dickenson, David L. H. Bennett

**Affiliations:** 1 Neuropharmacology of Pain Group, Department of Neuroscience, Physiology and Pharmacology, University College London, London, United Kingdom; 2 Neurorestoration Group, Wolfson CARD, King's College London, London, United Kingdom; University of Cincinnatti, United States of America

## Abstract

Joint degeneration observed in the rat monoiodoacetate (MIA) model of osteoarthritis shares many histological features with the clinical condition. The accompanying pain phenotype has seen the model widely used to investigate the pathophysiology of osteoarthritis pain, and for preclinical screening of analgesic compounds. We have investigated the pathophysiological sequellae of MIA used at low (1 mg) or high (2 mg) dose. Intra-articular 2 mg MIA induced expression of ATF-3, a sensitive marker for peripheral neuron stress/injury, in small and large diameter DRG cell profiles principally at levels L4 and 5 (levels predominated by neurones innervating the hindpaw) rather than L3. At the 7 day timepoint, ATF-3 signal was significantly smaller in 1 mg MIA treated animals than in the 2 mg treated group. 2 mg, but not 1 mg, intra-articular MIA was also associated with a significant reduction in intra-epidermal nerve fibre density in plantar hindpaw skin, and produced spinal cord dorsal and ventral horn microgliosis. The 2 mg treatment evoked mechanical pain-related hypersensitivity of the hindpaw that was significantly greater than the 1 mg treatment. MIA treatment produced weight bearing asymmetry and cold hypersensitivity which was similar at both doses. Additionally, while pregabalin significantly reduced deep dorsal horn evoked neuronal responses in animals treated with 2 mg MIA, this effect was much reduced or absent in the 1 mg or sham treated groups. These data demonstrate that intra-articular 2 mg MIA not only produces joint degeneration, but also evokes significant axonal injury to DRG cells including those innervating targets outside of the knee joint such as hindpaw skin. This significant neuropathic component needs to be taken into account when interpreting studies using this model, particularly at doses greater than 1 mg MIA.

## Introduction

Osteoarthritis (OA) is one of the most prevalent sources of chronic pain, affecting around 10% of men and 20% of women aged 60+ worldwide [1]. The disabling effect of osteoarthritis is not simply due to altered joint biomechanics, such as locking and crepitation, but also evoked and spontaneous pain associated with the arthritic joint [2]. Current analgesics are relatively ineffective and are associated with various gastrointestinal, cardiac and renal adverse effects [3].

The monoiodoacetate model of OA, in which a single injection of the irreversible NADPH inhibitor, sodium monoiodoacetate (MIA), is made into the joint space, provides a model of the painful and structural components of OA in rodents. The doses most frequently used are 1, 2 or 3 mg [4], [5], [6], with the model usually assessed up to 14 days post-induction, with some studies extending further to 30, 56 or 68 days [5], [7], [8]. MIA has been shown to inhibit chondrocyte metabolism, precipitating a rapid degeneration of joint integrity with features mirroring those seen clinically [5]. These include synovial thickening, loss of cartilage, formation of osteophytes and eventual fibrillation of cartilage. The inflammatory early phase of the model also features joint swelling and immune cell infiltration of the patellar fat pad, and resolves fully by day 7 [9], [10].

In parallel to degenerative changes within the joint, a pain phenotype rapidly develops in the hindlimb ipsilateral to the injected knee, suggesting the presence of central sensitization. This phenotype has been assessed using standard behavioural measures of evoked pain, including mechanical and thermal stimuli applied to an area of referred pain on the hindpaw, as well as calibrated pressure and torque applied to the knee [8], [11], [12]. Novel measures employed, intended to gauge movement evoked or ongoing pain, include tests of motility, weight-bearing, grip strength and sleep disruption [9], [10], [12], [13], [14], [15], [16], [17].

Electrophysiological studies have demonstrated peripheral changes in the excitability of knee joint afferents as well as central changes in the evoked responses and pharmacological manipulation of deep dorsal horn neurones with receptive fields in the hindpaw ipsilateral to joint degeneration [11], [18], [19]. These deep dorsal horn neurones are subject to increased 5-HT3R activity and increased endocannabinoidergic tone [18], [19].

While pregabalin, an analgesic clinically effective in a variety of neuropathic conditions [20], has only minimal effectiveness on deep dorsal horn neuronal evoked responses in sham animals, it is able to significantly reduce responses in MIA animals [19]. A similar state-dependency is seen in the action of a related drug with a similar mechanism of action, gabapentin, which is more effective in modulating nociceptive transmission in the presence of evoked central sensitization in human subjects [21].

MIA has been shown to evoke ATF-3 expression in DRG cells, which was suggested to represent damage to joint afferents [22]. Pain related hypersensitivity in this model has therefore been attributed to inflammation and degenerative changes within the knee joint, as well as a possible localised neuropathic component involving joint afferents.

Here we have characterised the expression of ATF-3 (a marker of neuronal injury), peripheral innervation and spinal microgliosis following MIA treatment of rats up to 14 days after model induction. We demonstrate significantly increased expression of ATF-3, primarily in L4 and L5 DRG, after intraarticular injection of 2 mg, but not 1 mg MIA. ATF-3 upregulation in the 2 mg MIA model is associated with reduced intraepidermal nerve fibre (IENF) innervation of ipsilateral plantar hindpaw skin, spinal microgliosis and greater hindpaw mechanical hypersensitivity, indicating a significant neuropathic component at this dose which includes afferents innervating territories outside of the knee joint. Additionally, while deep dorsal horn evoked responses are reduced by pregabalin in the 2 mg MIA model, the effects of the drug are significantly less pronounced where arthritis has been induced by 1 mg MIA.

## Materials and Methods

### Ethics Statement

All experimental procedures were approved by the UK Home Office (project license 1205) and follow the guidelines of the International Association for the Study of Pain [26].

### Animal care and model induction

Male Sprague-Dawley rats (Central Biological Services, University College London) weighing 120–140 g at the time of induction were used. Animals were anaesthetised using isofluorane and a single injection of 25 ul sterile 0.9% saline containing 2 mg or 1 mg of monosodium iodoacetate (MIA, Sigma, UK) was administered through the left patellar tendon using a 27G needle. Sham injections used ipsilateral saline only, and were assessed histologically at day 7, except for articular histology, which used contralateral saline and was assessed at day 14.

### Behaviour

Behaviour was assessed at 3, 7 and 14 days following MIA injection. Testing was preceded by a 30-min acclimatisation period. Cooling hypersensitivity was assessed using a drop of acetone applied to the plantar surface of the hind paws, both ipsilateral and contralateral to the injected knee. A marked, delayed, flinching or shaking behaviour was a positive outcome. The test was repeated a total of five times on each side with a minimum of 5 min between each application. The resulting score was doubled to give a score out of 10, allowing plotting on the same axes as mechanical hypersensitivity. Mechanical hypersensitivity was assessed by applying 1, 6 and 8 g von Frey filaments, (Touch-test, North Coast Medical Inc., San Jose CA, USA) 5 times to the plantar surface proximal to the digits, and 5 times to the toes of the ipsilateral and contralateral hind paws. Withdrawal responses and whole paw lifts elicited by von Frey hairs were scored as positive, with a Mean Difference Score of ipsilateral – contralateral withdrawals used for graphing and statistical analysis, which used Kruskall-Wallis test with Dunn's or Bonferroni's multiple comparison test for timecourse and between group comparisons respectively.

Weight bearing was assessed using an incapacitance tester (Linton Instrumentation, Norfolk, UK). Rats were placed in a plexiglass enclosure so that each hindpaw rested on a separate weighing plate. After 2 minutes' habituation, the force exerted by each hind paw was measured over a 5s testing period. The first 3 sets of 5s measurements were taken and then averaged. These values were then transformed to give the percentage of total hindlimb weight borne on the ipsilateral side. Data were analyzed using One-way ANOVA or 2-way ANOVA with Bonferroni's Multiple Comparison test for time course and between group comparisons respectively.

Experimenters were blinded to treatment groups where saline or different doses of MIA were used.

### Immunohistochemistry

Animals were terminally anaesthetised and transcardially perfused with chilled 4% paraformaldehyde in 0.1% phosphate buffer. Lumbar spinal cord was dissected out with ipsilateral and contralateral L3, L4 and L5 DRG and proximal sciatic nerves attached. For nerve morphometry studies, the sciatic nerve was followed until it branched into the tibial, common peroneal and sural nerves posterior to the knee joint (the tibial nerve includes the posterior articular nerve that innervates the articular structures). 5 mm samples were taken from each branch, proximal to the knee joint. Additional 5 mm samples were taken from the common peroneal nerves 10 mm distal to the knee joint. Skin biopsies were taken from the hindpaw glabrous skin proximal to the pad below the first digit, taking the full width of the plantar surface.

Tissues were postfixed in 4% paraformaldeyde for 2 hours at room temperature then transferred into 20% sucrose overnight at 4°C [27]. Tissue was embedded in OCT and stored at −80°C. Transverse sections of skin were cut at 14 µm on a cryostat onto chrome alum gelatin-covered slides. DRG and transverse spinal cord sections were cut at 10 µm on a cryostat onto SuperFrost Ultra Plus slides.

Nerves were postfixed in 3% glutaraldehyde at 4°C overnight, washed in 0.1 M PB, osmicated, dehydrated, and embedded in epoxy resin (TAAB Embedding Materials). 1 µm sections were cut on an ultramicrotome and stained with toludine blue.

After overnight postfixation in 4% PFA, knee joints were dissected to remove muscle and tranferred into a decalcifying buffer comprising 7% AlCl3, 5% formic acid, and 8.5% HCl for 10 hours at 4°C, before being washed in 0.1 M phosphate buffer, pH7.2. processed and embedded in paraffin wax. 6 µm sagittal sections from each condyle were cut and mounted onto microscope slides, then dried at 60°C overnight. After dewaxing and rehydrating, sequential sections were stained using Toluidine blue pH4 to visualize proteoglycans in the articular cartilage.

DRG cells were visualized by immunostaining with antibodies raised against βIIITubulin (Promega, mouse monoclonal, 1∶800) and ATF-3 (Santa Cruz, rabbit polyclonal, 1∶400). Intra-epidermal nerve fibres (IENF) in skin biopsies were visualized with the pan-neuronal marker protein gene product (PGP), polyclonal rabbit PGP9.5 (Ultraclone, 1∶800). Spinal cord microglia were visualized using rabbit anti-Iba1 (WAKO, 1∶1000). Secondary antibodies used were anti-rabbit Cy3 (Stratech; 711-166-152; 1∶500; 2.5 hr) and AlexaFluor 488 anti-mouse (Invitrogen, 1∶1000, 2.5 hr). Overnight incubation of primary antibodies was preceded by incubation with normal donkey serum (Millipore Bioscience Research Reagents; S30; 1∶10; 30 min). All reagents were diluted in PBS/0.2% Triton X-100/0.1% sodium azide.

Immunofluorescence was visualized under a Zeiss Imager.Z1 microscope.

Four mosaic-photomicrographs of complete DRG sections per DRG were randomly selected from each animal. Both ATF-3^+^ and the total BIIITub^+^ nucleated cell profile populations were counted and their diameters measured to determine cell size distribution. Because on sectioning DRG cells will be cut into multiple profiles we refer to DRG cell profiles and not absolute DRG cell numbers when discussing quantification of ATF-3 expression. Cell counting and analysis was performed using AxioVision LE, release 4.2.

Epidermal fibers were counted at 40× magnification live on the microscope according to rules set out by Lauria et al. [28]. Only nerve fibers that could be seen to cross the basement membrane between the epidermis and dermis were counted. Four skin sections were counted per animal, by a blinded experimenter. The length of the epidermis sample in mosaic-photomicrograph was measured using ImageJ and the intraepidermal nerve fiber density (IENFD) calculated as number of fibers per millimeter. Data were analyzed using paired t-test of ipsilateral vs contralateral IENF density, or unpaired t-test of 2 mg vs 1 mg IENF ipsilateral density as a percentage of contralateral density.

Quantification of the number and morphology of Iba1 immunoreactive microglia in the spinal cord was performed in four sections of spinal cord at the level of L4 per animal. Microglia were quantified within 4 defined 10,000 um^2^ areas in the superficial dorsal horn or ventral horn. Microglia in which process length was less than double the soma diameter were classified as presenting an effector morphology, while microglia in which process length was more than double soma diameter were defined as possessing surveyor morphology, a method used previously by [29]. Cells were sampled only if the nucleus was visible within the plane of section and if cell profiles exhibited distinctly delineated borders. Spinal cord ATF-3 staining was quantified as the total number of ATF-3^+^ profiles present in 4 spinal cord sections per animal.

Knee joint sections were imaged using an Epson perfection V700 photo flatbed scanner at 3200 dpi. Cartilage proteoglycan content was scored on a scale of 0–4 where 0 = no loss of cartilage proteoglycan staining relative to a normal control, 1 = minimal loss, 2 = mild loss, 3 = moderate loss and 4 = total loss of proteoglycan staining. The extent of cartilage proteoglycan loss was assessed as 1/3, 2/3 or 3/3 of the condylar surfaces and the above score was multiplied by 1, 2 or 3, respectively to give a maximum score of 12 for total loss over the whole surface [30]. For articular histology only, ipsilateral MIA treated knees were compared to contralateral saline-injected knees.

In all cases, quantification was performed by a single, blinded observer. Unless stated otherwise, data were analysed using One-way ANOVA with Dunnett's or Bonferroni's multiple comparison test for timecourse or between-group comparisons respectively.

### Electrophysiology

Two weeks after MIA injection (post-operative days 15–19), in vivo electrophysiological studies were performed as previously described [31]. Briefly, animals were anesthetized and maintained for the duration of the experiment with isofluroane (1.5–1.7%) delivered in a gaseous mix of N2O (66%) and O2 (33%). A laminectomy was performed to expose the L4–5 segments of the spinal cord. Extracellular recordings were made from ipsilateral deep dorsal horn neurones (laminae V–VI) using parylene coated tungsten electrodes (A–M Systems, USA). The neurones included in this study met the following criteria: they had a receptive field on the plantar hindpaw; they all responded with at least 50 spikes to both light touch (8 g von frey) and noxious stimuli (60 g von Frey and 48°C heat); they responded to natural stimuli in a graded manner with coding of increasing intensity; they exhibited windup when repeatedly stimulated; and they were situated at a depth of >500 µm from the surface of the spinal cord.

A train of 16 transcutaneous electrical stimuli (2 ms wide pulses, 0.5 Hz) applied at 3 times the threshold current for C-fibre activation of the dorsal horn cell was delivered via stimulating electrodes inserted into the peripheral receptive field in the hindpaw. A post-stimulus time histogram was constructed such that responses evoked via Aβ – (0–20 ms), Aδ – (20–90 ms) and C-fibres (90–350 ms) were separated and quantified on the basis of latency. Responses occurring after the C-fibre latency band were taken to be the post-discharge (repetitive firing) of the WDR cell (350–800 ms).

The centre of the peripheral receptive field was also stimulated using punctate mechanical and thermal stimuli (2, 8, 26 and 60 g von Frey filaments and a water jet applied at 35, 40, 45, and 48°C). Application of each von Frey hair was separated by a minimum interval period of 50 seconds. All natural stimuli were applied for a period of 10 seconds per stimulus. Data was captured and analysed by a CED 1401 interface coupled to a computer running Spike 2 software (Cambridge Electronic Design; PSTH and rate functions).

Pharmacological assessment was carried out on one ipsilateral neuron only per animal. One round of testing was performed every twenty minutes, and consisted of a train of electrical stimuli followed by graded natural stimuli as described above. Following three consecutive stable control trials (<10% variation for the C-fibre evoked response) neuronal responses were averaged to give the pre-drug control values.

Pregabalin (a gift from Pfizer, Sandwich, UK) was dissolved in 0.9% saline solution at a concentration of 10 mg/kg, and administered via subcutaneous injection in the scruff of the back of the neck. Previous studies indicated that this concentration significantly reduced spinal neurone evoked responses in 2 mg MIA treated, but not sham injected, rats [19]. The effect of the drug was followed for an hour, with tests carried out at 10, 30 and 50 minutes after dosing. The value of greatest change from the baseline for each metric (electrical stimuli, natural stimuli) was then found and expressed as a percentage of the predose baseline, and plotted to allow comparison of drug effect in sham, 1 mg MIA and 2 mg MIA animals. Kruskal-Wallis tests with Dunn's posttest were used to compare drug effect for each metric. The sham and 2 mg data is included for comparison, but has previously been published in a different form [19].

## Results

### Pain behaviour and cartilage loss following intra-articular MIA treatment

**Figure 1:**
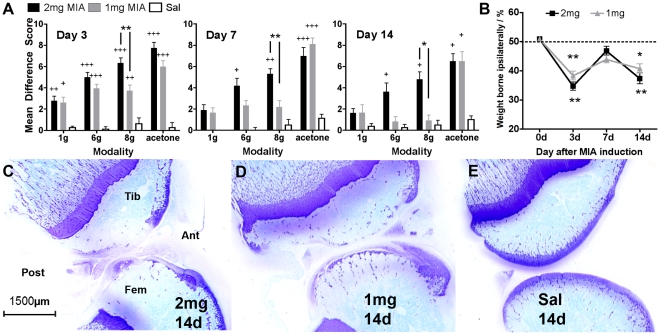
Behavioural and articular histological assessment of MIA animals. A – withdrawals in response to 1 g, 6 g or 8 g von frey hair or acetone applied to the plantar hindpaw 3, 7 or 14 days after intra-articular saline, 2 mg or 1 mg MIA injection (Kruskal Wallis test of: timecourse, P<0.05 +, P<0.01 ++, P<0.001 +++ vs preinjection baseline; inter-group comparison P<0.05 *, P<0.01 ** n = 12 animals/group). B – incapacitance testing of 2 mg or 1 mg MIA treated animals at 3, 7 and 14d after treatment (2-way ANOVA with Bonferroni's post test, n = 12 animals/group. P>0.05 at all time points). C – 2 mg MIA treated rat knee sagittal sections from day 14, stained with toluidine blue to visualise cartilage proteoglycan content. Fem = femoral condyl, Tib = tibial condyl. Ant = anterior aspect of knee, Post = posterior aspect. D – 1 mg MIA rat knee sections from day 14. E – saline injected contralateral control knee from day 14.

Arthritis-associated referred pain behaviours were assessed at the hindpaw as previously described [13]. Significant hypersensitivity to von Frey hair application and acetone cooling were both observed by day 3 in 2 mg and 1 mg MIA animals (Figure 1A Kruskal Wallis test, P<0.05 vs preinjection baseline, n = 12 animals/group). Significant weight bearing difference between ipsilateral and contralateral hindlimb was seen following 2 mg and 1 mg MIA injection (Fig. 1B, One-way ANOVA with Dunnett's multiple comparison test, P<0.05, n = 12 animals/group). There was no hypersensitivity on the contralateral hindpaw. Consistent with previous studies, the timecourse of weightbearing asymmetry is biphasic, with asymmetry slightly correcting at the 7d timepoint but returning at 14d [6], [10]. The weight-bearing alteration in the 2 mg group was not significantly different to that in the 1 mg group (2-way ANOVA with Bonferroni's post test, n = 12 animals/group, P>0.05 at all time points).

When comparing the 1 mg and 2 mg MIA groups there was significantly less mechanical hypersensitivity in the 1 mg group, with significantly fewer withdrawals to the 8 g von Frey hair at all time points compared to the 2 mg group (Fig. 1A, Kruskal Wallis test with Bonferroni's posttest, P<0.05). No significant difference between 1 mg and 2 mg groups was apparent in the response to acetone cooling or weight bearing at any timepoint.

Despite the difference in evoked behaviour, the extent of articular cartilage proteogylcan loss at 14d was not significantly different in 2 mg (Fig. 1C) vs 1 mg (Fig. 1D) groups (Table S1), consistent with existing data at this time point [32]. Note that, after the 7d MIA stage, there is no significant inflammation in the joint structures [9]. Because the degree of cartilage loss could not explain the difference in mechanical hypersensitivity when comparing the 2 and 1 mg dose of MIA at day 14, we went on to assess the extent of peripheral neural injury.

### Increased ATF-3 expression within DRG cells and motoneurones following MIA application

**Figure 2:**
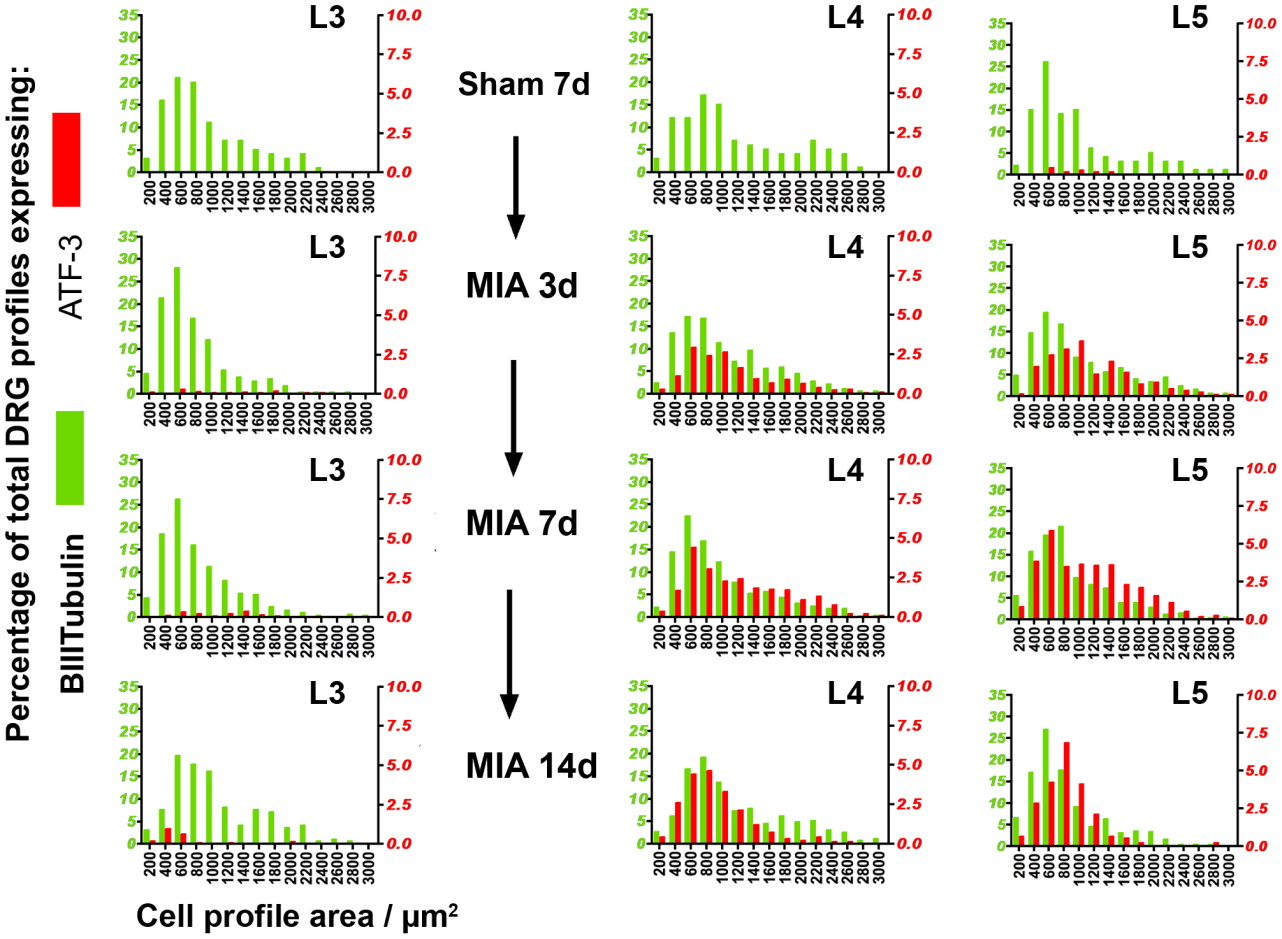
Cell size distribution for BIIITubulin (green bars, left y axis) and ATF-3 (red bars, right y axis) expressing profiles in DRG L3, L4 and L5 in sham or 2 mg MIA animals 3, 7 and 14 days after injection. n = 9 sham, 7 at day 3, 12 at day 7, 7 at day 14.

**Figure 3:**
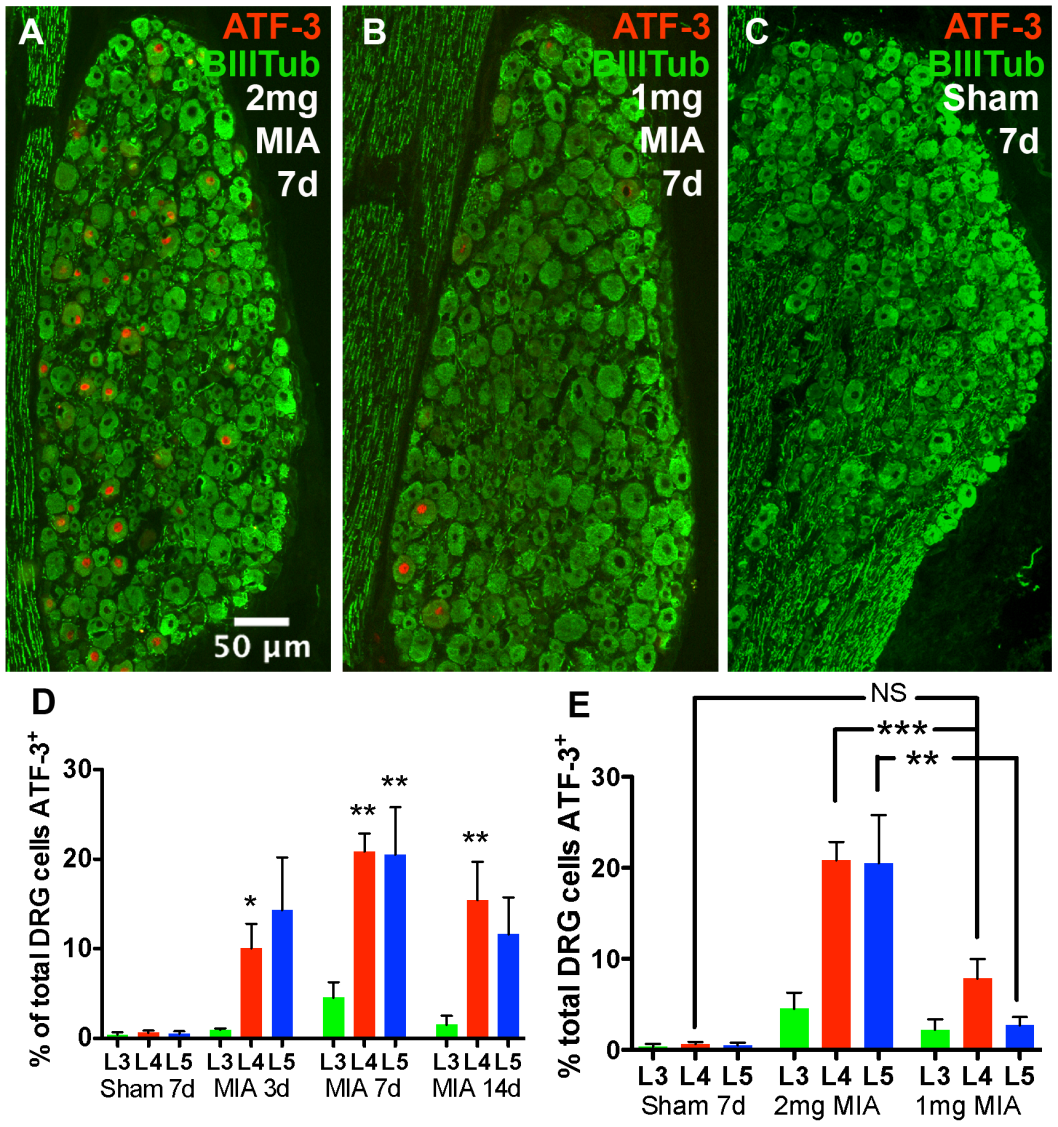
Approximation of total DRG ATF-3 expression. A, B, C - DRG profiles show nuclear expression of ATF-3 7 days after 2 mg (A) or 1 mg (B) MIA, but not 14 days after saline sham injection (C). D - Estimated fraction of DRG profiles expressing ATF-3 in 2 mg MIA-treated animals (One-way ANOVA with Dunnett's multiple comparison test, n = 9×14d sham, 7×3d, 12×7d MIA, 7×14d MIA, P<0.01 **, P<0.05 *). E - Comparison of ATF-3 expression in 2 mg vs 1 mg MIA injected groups at 7 day timepoint (1 way ANOVA with Bonferroni's multiple comparison test, n = 9×7d sham, 12×2 mg MIA, 12×1 mg MIA, P<0.01 ** P<0.001 ***).

The expression of the transcription factor ATF-3 is a sensitive marker of neuronal insult or injury. It is expressed in DRG cells and spinal cord ventral horn motoneurons within 3d days following axotomy or crush, remaining positive for at least 28 days after nerve injury [33], [34]. Immunohistochemistry for ATF-3 and BIIITub positive cell profiles in the DRG enabled approximation of the percentage of total DRG cell nuclear profiles immunopositive for ATF-3 at the level of L3, L4 and L5 3, 7 and 14 days after MIA injection (Figure 2, illustrated and quantified as % of DRG in Figure 3A). Analysis of cell size distribution showed ATF-3 expression was not restricted to one population of cells but rather present in small, medium and large cell profiles.

The total mean ATF-3 signal peaked at 20.83% of nucleated profiles quantified in L4 at day 7 (Fig. 3D). Total mean ATF-3 signal was below 5% in L3 at all time points. No ATF-3 positive nuclear profiles were present in L2 and L6 DRG (data not shown). Sham injection of saline did not induce significant ATF-3 expression at any of the levels assessed at day 7, though ATF-3 expression in naïve DRG was observed very infrequently (Figure 3C, D, in <1% of profiles counted). ATF-3 expression was significantly greater at 7 and 14 days after MIA injection compared to 7d sham in L4 (Figure 3D, One-way ANOVA with Dunnett's multiple comparison test, n = 9×7d sham, 12×7d MIA, 7×14d MIA), and at 7 days after injection in L5 (n = 12×7d, P<0.01).

As expression in the 2 mg group peaked at the 7d timepoint, this timepoint was chosen to assess ATF-3 expression in the 1 mg MIA group. ATF-3 expression in 1 mg MIA treated rats (Figure 3B) was not present at a level significantly greater than that seen in saline injected sham animals at the 7d timepoint, although a strong trend was noted. There were significantly fewer ATF-3^+^ profiles in 1 mg (7.6% of L4 DRG) than in 2 mg (20.83% of L4 DRG) MIA treated animals at the 7 day timepoint (Figure 3E, 1 way ANOVA with Bonferroni's multiple comparison test, n = 9×7d sham, 12×2 mg MIA, 12×1 mg MIA, 2 mg vs 1 mg P<0.001 at L4, 2 mg vs 1 mg P<0.01 at L5, 7d sham vs 1 mg P>0.05 at L4 or L5).

Immunohistochemistry also indicated the presence of ATF-3 positive nuclei in ventral horn motor neurones of 2 mg MIA treated animals (Figure S1). The absolute number of these ATF-3 positive nuclei was small (mean of 1.2 ATF-3^+^ cells per section of L4 spinal cord at 7d after MIA injection), but was significant at day 7 (one-way ANOVA with Dunnett's multiple comparison test, n = 8 animals per timepoint, p<0.05). ATF-3 positive ventral horn nuclear profiles were not present in the 1 mg MIA or 7d sham treated groups.

The cell bodies of afferent fibres innervating the joint reside in primarily L3 and L4 DRG [35], and comprise below 5% of the total cell bodies in the DRG at these levels [36]. As the magnitude of ATF-3 expression observed in L5 DRG could not arise purely from joint afferents, we studied nerve morphometry as well as primary afferent terminations within hindpaw plantar epidermis.

### Intra-articular MIA is associated with a reduction in ipsilateral hindpaw intra-epidermal nerve fibre density

Because the sciatic nerve branches into the tibial, peroneal and sural nerves posterior to the knee joint we examined toluidine blue stained semithin sections of these nerves (Figure S2). There was no evidence of structural degeneration, demyelination, inflammatory infiltrate or any other difference between ipsilateral and contralateral samples at any of the sites assessed at day 7 following 2 mg MIA induction.

**Figure 4:**
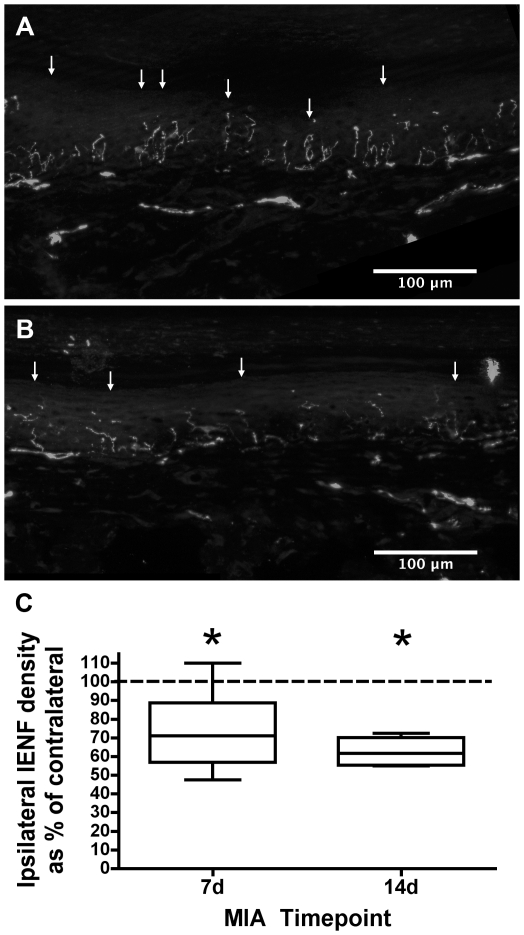
Quantification of intrapepidermal nerve fibre density in plantar hindpaw following MIA treatment. A, B - sections of naïve (A) and MIA (B) plantar hindpaw skin containing PGP9.5 immunoreactive intraepidermal nerve fibres (arrows). C – quantification of IENFs at the 7 and 14 day timepoints (paired t-test of ipsi vs contralateral density, n = 8×7d, 4×14d, P<0.05). IENF density reduction is not seen in 1 mg MIA animals.

To investigate the possibility of a ‘dying back’ small fibre neuropathy, intra-epidermal nerve fibre density (IENFD) was quantified in the hindpaw. This measure is often used clinically and experimentally [27], [37] to assess small fibre integrity in peripheral neuropathies. PGP9.5 immunoreactive IENFs were quantified in skin sections from the ipsilateral and contralateral plantar hindpaw in a blinded fashion (Figure 4A, B). 2 mg MIA induction was associated with a significant decrease in ipsilateral IENFD of 26% at day 7 and 37% at day 14 (Figure 4C, paired t-test of ipsi vs contralateral density, n = 8×7d, 4×14d, P<0.05).

In 1 mg MIA treated rats there was no evidence of ipsilateral reduction in IENFD (P>0.05 vs contra side, paired t test). The ipsilateral IENFD was significantly higher than the ipsilateral 2 mg IENF density at the same time point (unpaired t test, P<0.01, n = 8×7d 2 mg, 4×7d 1 mg).

### Intra-articular MIA is associated with spinal microgliosis

**Figure 5:**
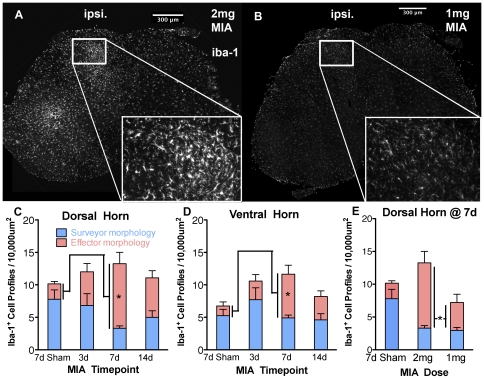
Quantification of microglial activation in spinal cord following MIA treatment. A, B - expression of the microglial marker iba1 in L4 spinal cord in 2 mg (A) and 1 mg (B) MIA groups. C, D – Quantification of microglia with effector morphology in dorsal (C) and ventral (D) horn (1-way ANOVA, n = 4×7d sham, 11×2 mg MIA, P<0.05 *). E – Comparison of dorsal horn microgliosis in 2 mg vs 1 mg MIA groups at the 7d timepoint (1-way ANOVA, n = 4×7d sham, 11×2 mg MIA, 8×1 mg MIA, P<0.05 *).

In a wide range of models, nerve injury is associated with increased numbers of microglia within the spinal cord [38], [39]. Treatment with intra-articular MIA at a dose of 2 mg resulted in microgliosis within both the dorsal and ventral horn as demonstrated by immunohistochemistry for the microglial marker Iba1 (Figure 5A). Sham rats, which received intra-articular saline, had no microgliosis at day 7 and were indistinguishable from naives. Microgliosis produced by 2 mg MIA treatment was associated with a significant shift to *effector* morphometry, both dorsally and ventrally, by day 7 compared to sham (Figure 5C and D, One-way ANOVA with Dunnett's multiple comparison test, n = 4×7d sham, 11×2 mg MIA, P<0.05). In 2 mg MIA rat ventral horn, cells were observed enwrapping motor neuronal cell bodies. Microgliosis was noted at levels L3–L5, but was most consistent at L4, where quantification was performed. GFAP immunoreactivity was quantified in each spinal cord quadrant to assess astrogliosis, but showed no significant change (data not shown).

As iba-1 reactivity peaked at the 7d timepoint, this timepoint was selected to assess 1 mg MIA group microglial activity. Following 1 mg treatment the total number of microglia counted was not significantly greater than in saline injected 7d sham animals, and was significantly less than the count in 2 mg MIA rat dorsal horn (Figure 5B). Although there was a trend for increased change in morphology in the 1 mg group, this was not significant (Figure 5E, One-way ANOVA with Bonferroni's multiple comparison test, n = 4×7d sham, 11×2 mg MIA, 8×1 mg MIA, P>0.05 1 mg vs 7d sham, P<0.05 2 mg vs 1 mg).

### Systemic Pregabalin Reduces Deep Dorsal Horn Evoked Responses in 2 mg, but not 1 mg MIA treated animals

Extracellular action potentials were recorded from deep dorsal horn neurones with receptive fields on the plantar hindpaw, where behavioural hyperresponse to normally innocuous stimuli is seen in MIA animals (as above). These neurones were characterised as wide dynamic range cells (WDR), in that they gave a graded response to a wide range of innocuous and noxious mechanical and thermal stimuli, exhibited windup, and were located in laminae V–VI of the dorsal horn, as previously described [40].

**Figure 6:**
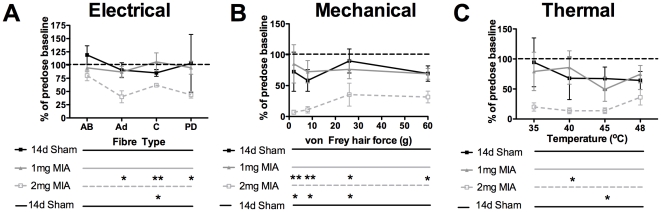
Effect of pregabalin treatment (10 mg/kg s.c.) on evoked responses of deep dorsal horn WDR neurones. A - Electrically evoked responses in the Aß, A∂ and C fibre range, as well as PD (post-discharge/repetitive firing) expressed as % of predose baseline in 2 mg MIA, 1 mg MIA or sham injected animals. B - Mechanically evoked responses expressed as % of predose baseline in 2 mg MIA, 1 mg MIA or sham injected animals. C - Thermally evoked responses expressed as % of predose baseline in 2 mg MIA, 1 mg MIA or sham injected animals. Note the significantly greater effectiveness of pregabalin in the 2 mg treated group (normalized post-drug responses compared using Kruskal Wallis test with Dunn's posttest. n = 14d sham×9, 1 mg MIA×8, 2 mg MIA×9. P<0.05 *, P<0.01, **). The sham and 2 mg data is included for comparison, but has previously been published in a different form [19].

As shown previously [19], pregabalin has minor inhibitory effects on evoked responses in animals that underwent an intraarticular saline sham injection 14 days before recording (Figure 6A, B and C black traces show drug effect on electrical, mechanical and thermal evoked responses, respectively). WDR cells in animals that received 2 mg MIA were significantly more sensitive to pregabalin inhibition (Figure 6A, B, C grey dotted trace). In contrast, pregabalin inhibition in animals that received 1 mg MIA was not significantly different to sham for any of the evoked responses assessed (Figure 6A, B, C grey solid vs black traces). Pregabalin inhibited electrically evoked responses in the C fibre range and 8 g and 60 g evoked mechanical responses to a significantly greater extent in 2 mg MIA treated animals than in 1 mg treated animals (Figure 6A, B, Kruskal-Wallis test with Dunn's post test, n = sham×9, 1 mg MIA, ×8, 2 mg MIA×9).

## Discussion

In this study we have carefully characterised the widely used MIA model of OA in relation to the development of pain related behaviour, the degree of joint cartilage loss and neural injury, and the extent of dorsal horn pharmacological plasticity. To summarise, the higher dose of MIA (2 mg) was associated with greater hindpaw mechanical hypersensitivity than the lower dose (1 mg) treatment. However there was no difference in the degree of cartilage proteoglycan loss. 2 mg, but not 1 mg, MIA produced an increase in the expression of the injury marker ATF-3 in DRG cells, a reduction in intra-epidermal nerve fibre density within plantar epidermis, and ipsilateral spinal cord microgliosis, indicating significant neural injury. Systemic pregabalin significantly inhibited deep dorsal horn WDR cell evoked responses in 2 mg MIA treated animals, but not in 1 mg- or sham treated animals.

In studying pain-related behaviour, previous investigators have used a range of doses from 1 to 4.8 mg MIA. We elected to use either 1 or 2 mg, at the lower end of this dose range. Dose-dependent effects of MIA have been demonstrated for weight-bearing asymmetry, cartilage biosynthetic processes and bone density loss [6], [10]. These effects tend to plateau quickly at doses above 1 mg and indeed we found no difference in cartilage proteoglycan loss comparing 2 and 1 mg MIA; the behavioural measures of weight-bearing asymmetry and the response to acetone cooling were also not significantly different. However mechanical pain related hypersensitivity of the hindpaw was strikingly increased at the 2 mg versus 1 mg dose.

We used the expression of ATF-3 [33], [34] to assess the degree of injury to peripheral neurons following intra-articular MIA injection, in order to explore the possibility that differential degrees of neuronal injury may underlie the differing evoked behavioural phenotypes in the 1 mg and 2 mg MIA groups. One previous study has shown increased expression of ATF-3 in lumbar DRG cells using 1 mg MIA treatment, however these findings were not expressed in a manner in which it was possible to gauge the proportion of all DRG cells expressing ATF-3, and focussed more on timepoints after 14d [22].

There were a number of aspects of the ATF-3 response indicating that injury following intra-articular MIA is not restricted to joint afferents. The proportion of DRG cell profiles expressing ATF-3 was highest in L4 and 5, DRGs which principally project to the hindpaw, rather than L3 and 4, which contain the majority of joint afferents [35]. The size of the ATF-3 signal peaked at ∼20% of L4/L5 DRG profiles and cannot therefore have arisen solely from damage to the relatively small population (4.6%) of DRG intra-articular afferents, although it may include these afferents [36]. The lower dose of 1 mg MIA was associated with ATF-3 expression in much lower percentage of DRG cell profiless (7.6% of L4).

The signal seen in the MIA model could be the result of a direct action of MIA on the peripheral nerves running adjacent to the knee, perhaps secondary to leakage of MIA from the intra-articular space. We are highly experienced in this model and every effort is made to prevent any leakage of MIA at the time of intra-articular injection. Retrograde tracers used in MIA rat joint actually label a smaller proportion of DRG cells compared to those traced from a normal joint [36] implying that it is not the case that joint capsule disruption during the course of the model could cause MIA to diffuse to a more widespread range of tissues. We do not see any evidence of systemic effects of MIA and the increased ATF-3 expression was always strictly unilateral, as were other markers of neuronal injury discussed below. MIA is able to induce massive Ca^2+^ influx in rat peripheral nerve *in vitro* via inhibition of metabolism, as well as being cytotoxic to non-neuronal cells such as astrocytes [41], [42].

Intra-articular MIA triggers a vigorous inflammatory response up to day 5 post-injection, with associated monocyte and neutrophil infiltration and joint swelling [5], [9]. A similar degree of joint swelling is observed in other inflammatory models of monoarthritis, meaning that swelling alone is unlikely to account for the greater ATF-3 signal observed in the MIA model [45]. It has recently been shown that some algogens, such as formalin and capsaicin, which strongly activate subsets of primary afferents when injected into the plantar hindpaw, trigger ATF-3 expression in DRG cells [43]. This raises the point as to whether inflammatory mediators released as a consequence of intra-articular MIA could contribute to increased ATF-3 expression.

ATF-3 expression has now been assessed in a number of different models of inflammation all of which are associated with enhanced pain-related behaviour. But neither intra-plantar application of Complete Freund's Adjuvant, antigen induced arthritis nor collagen-induced arthritis could induce ATF-3 expression above the level of ∼2% in lumbar DRG [44], [45].

Additionally, although inflammatory measures resolve by day 7 in the MIA model [10] ATF-3 signal is still present at day 14. This would appear to rule out a link between ATF-3 signal and inflammation. A further assessment of ATF-3 expression, a well as the other measures used in this study, at later timepoints such as 28 days after injection, would illuminate whether the pathological changes observed here persist, exacerbate or reverse with time. However, the existence of a robust non-inflammatory pain behavioural phenotype at the 14d timepoint, which neither exacerbates nor resolves over succeeding weeks [5], as well as the well characterised spinal pharmacological plasticity at this stage [18], [19] justify the focus of the present study on this timepoint.

Because of the strong ATF-3 signal in response to MIA in L4 and 5 DRG we studied the effects of this treatment on the innervation of hindpaw skin, a major peripheral target of these neurones. Quantification of intra-epidermal nerve fibre density (IENFD) is a sensitive means of assessing injury to small fibres used in both clinical practice [37] and animal models of toxic neuropathies [46], [47], [48], [49]. 14 days after intra-articular 2 mg MIA treatment, there was a 38% reduction in IENF density of hindpaw skin. In contrast, treatment with 1 mg MIA did not reduce IENFD.

This reduction in IENFD is similar to that previously reported in models of painful toxic neuropathies, for instance the 24–44% reduction seen in paclitaxel- and vincristine- experimental painful neuropathies [46]. There are broad similarities in the behavioural and anatomical phenotypes in 2 mg intra-articular MIA and experimental paclitaxel-neuropathy. Both share hindpaw mechanical and cooling, but not thermal, hypersensitivity, despite IENF loss, features common to many painful neuropathies [48], [50].

Traumatic nerve injury models are associated with a response in dorsal horn microglia, accompanied by subsequent astrocytosis, which may contribute to the development and maintenance of neuropathic pain [51], [52], [53]. In response to injury, microglia proliferate [29], [54], [55], migrate to the site of injury [56], [57] and undergo a morphological change to become less ramified and more amoeboid [58]. In terms of function, they can phagocytose cellular debris [59], present antigens [60] and secrete a broad range of cytokines and chemokines which amplify the transmission of nociceptive information in the dorsal horn [51]. The induction of microgliosis appears dependent on primary afferent electrical activity, as well as numerous neuron-to-glia signalling molecules including neuregulin-1, CCL2 and fractalkine [29]

Following 2 mg intra-articular MIA, we noted increased numbers of ‘effector’ microglia within both the dorsal and ventral horn. The 1 mg MIA treatment did not increase the total number of microglia within the dorsal horn nor lead to a significant change in their morphology. Previous studies have shown that the microglial response within the dorsal horn is robustly evoked by traumatic nerve injury but that certain models of chemotherapy-evoked peripheral neuropathy [61] and most inflammatory pain models [62], [63] do not produce a microglial response, with some exceptions [64]. This suggests that there may be a threshold of stress or axonal injury that a neuron needs to achieve before microglia can be recruited into a pro-inflammatory response, even when a ‘dying back’ neuropathy of sensory neurones is present. The presence of microglial activation at the 14d timepoint, while inflammation resolves at the 7d timepoint [9], also suggests that microglial activation in this case is not primarily linked to inflammation

Numerous studies indicate that gabapentinoid drugs, such as pregabalin, are significantly better able to modulate spinal innocuous and nociceptive transmission in models of neuropathy or other pain states than under physiological conditions [19], [65], [66]. This is supported clinically by functional magnetic resonance imaging evidence that gabapentin is significantly better able to modulate nociceptive transmission following the induction of central sensitization [21]. The ability of pregabalin to reduce deep dorsal horn evoked responses significantly in the 2 mg MIA group, but not in 1 mg MIA or sham treated animals, may thus indicate the presence of central sensitization in the 2 mg group that is not present in the 1 mg animals. This may account for the reduced behavioural hypersensitivity to stimuli applied to the hindpaw referred-pain area in 1 mg MIA treated animals compared to more sensitized 2 mg treated group. Alternatively, the differential effect of pregabalin could reflect the differing extents of peripheral neuronal injury in the 2 mg and 1 mg groups.

It should be noted that while the presence of central sensitization is often found to be a necessary condition for gabapentinoid efficacy, it is clearly not a sufficient condition. Hence, although central sensitization may be present in some other inflammatory chronic pain states, in the absence of additional factors (such as the presence of neuropathy, or activation of descending serotonergic facilitatory controls) gabapentinoid efficacy does not increase [19], [65].

Clinical studies suggest the existence of a subpopulation of OA patients who experience pain with neuropathic features. In patients undergoing surgical management of OA, pain often persists after joint replacement [23]. A recent imaging study reveals abnormal descending controls from brain to spinal cord in a subgroup of patients with OA [24], an event seen in animal neuropathic models as well as in the MIA model [19], [25], with the extent of this change correlating with patients' use of neuropathic pain descriptors. Coupled with the demonstration of increased central sensitization and referred pain [67], these studies suggest the existence of central changes in OA resembling those seen in neuropathic pain.

Furthermore, in addition to reported cutaneous hypoaesthesia with paradoxical mechanical allodynia overlying the degenerating knee in OA patients, there is also loss of peripheral vibratory sense distal to the degenerating knee in these patients, with the degree of loss of acuity proportional to the radiographic OA severity [68], [69], [70]. Whether this loss correlates to any alteration in IENF in these distal territories clinically is currently unknown.

There is considerable variation in the dose and volume used preclinically to induce the MIA model. Assessment of 43 studies published to date indicates that 1, 2 and 3 mg MIA doses were in equally common usage (Table S2). The available data suggests that there are different pathophysiological sequellae that follow different doses of MIA, with the 2 mg dose associated with a much greater degree of neuronal injury and/or central sensitization than 1 mg MIA. This includes a ‘dying back’ of small fibre afferents innervating targets outside of the knee joint, which, if it can be attributed to neurotoxicity, that would call into question the utility of the 2 mg MIA model for translational study of osteoarthritis pain. However, the concurrent neuropathic and osteoarthritic phenotypes of the 2 mg MIA model, irrespective of the etiology of the former, could still allow the use of this model to investigate clinical osteoarthritic pain with neuropathic features/descriptors

In summary, the divergent sequellae following different doses of MIA must be taken into account when interpreting data generated using the model, especially where indirect outcome measures of joint pain have been employed and where doses greater than 1 mg MIA are used.

## Supporting Information

Figure S1 **Spinal cord expression of ATF-3 following 2 mg MIA treatment.** A - nuclear expression of ATF-3 in magnocellular ventral horn ipsilateral to 2 mg MIA injection. B - quantification of total ATF-3^+^ nuclei/4 sections counted (one-way ANOVA with Dunnett's multiple comparison test, n = 8 animals per timepoint, p<0.05). 1 mg MIA was not associated with ventral horn ATF-3 expression at day 7.
(TIF)

Figure S2 **Analysis of peripheral nerve following 2 mg MIA injection.** A, B - toluidine-blue stained semithin sections of common peroneal nerve ipsilateral (A) or contralateral (B) to 2 mg MIA injection at day 7 of the model. There is no visible axon degeneration, demyelination or inflammatory cell infiltrate (n = 4 per group).
(TIF)

Table S1 **Mean cartilage proteoglycan score for 2 mg MIA, 1 mg MIA and saline injected joints, as assessed using toluidine blue staining (See** **Fig. 1C, D** **.).** Scoring reflects the degree of proteoglycan loss, with 12 corresponding to total loss over the entirety of both condylar surfaces, while 1 is undisrupted cartilage proteoglycan. n = 4×1 mg, 3×2 mg animals, +14d saline injected contralateral controls.
(DOC)

Table S2 **43 published studies which used the MIA model.** Columns denote dose of monoiodoacetate used. The highest dose was 4.8 mg, the lowest was 0.01 mg. Studies in bold specifically assessed evoked pain responses at the hindpaw. Where multiple doses were used, a study is referenced at each dose.
(DOC)
