# Supplementary figures and images for: Characterisation of a Peripheral Neuropathic Component of the Rat Monoiodoacetate Model of Osteoarthritis
Source: PLoS One. 2012 Mar 21;7(3):e33730. doi: 10.1371/journal.pone.0033730 (PMC3312347; doi:10.1371/journal.pone.0033730)

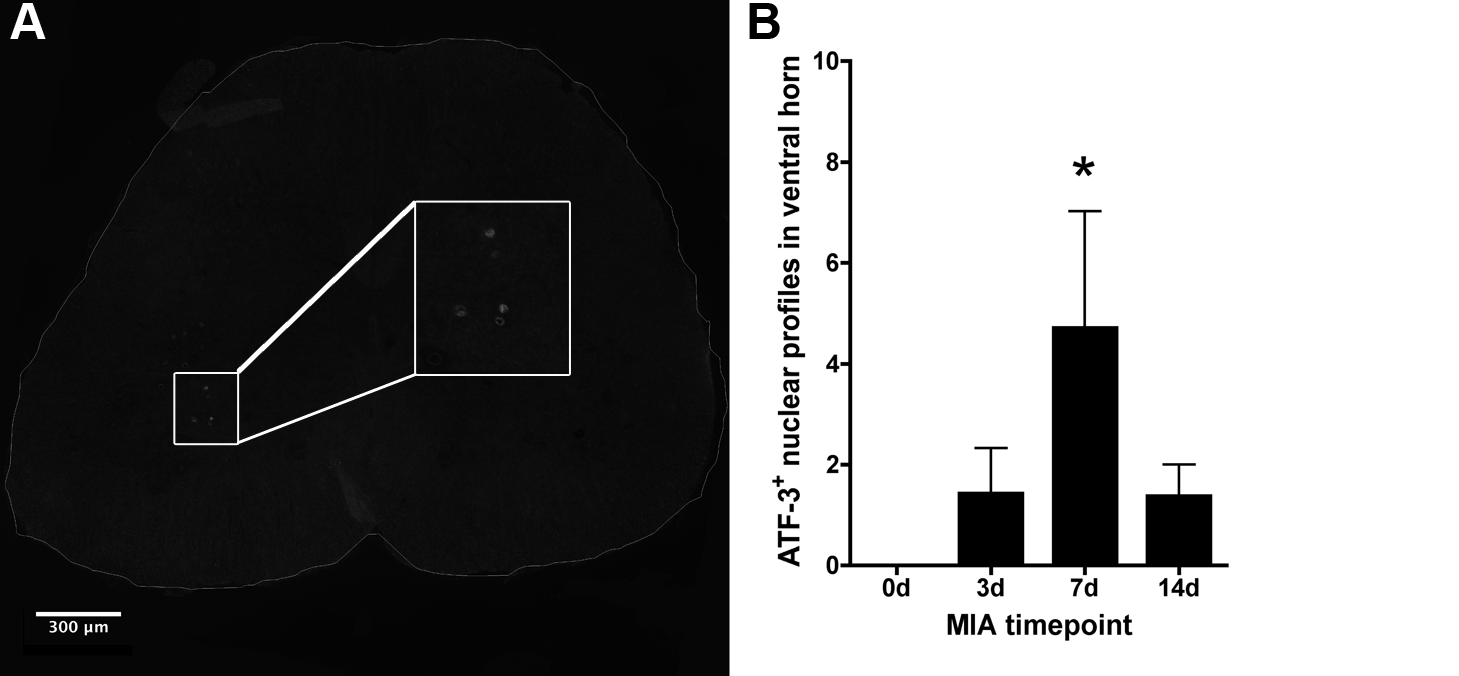

Supplement: Figure S1 — Spinal cord expression of ATF-3 following 2 mg MIA treatment. A - nuclear expression of ATF-3 in magnocellular ventral horn ipsilateral to 2 mg MIA injection. B - quantification of total ATF-3+ nuclei/4 sections counted (one-way ANOVA with Dunnett's multiple comparison test, n = 8 animals per timepoint, p<0.05). 1 mg MIA was not associated with ventral horn ATF-3 expression at day 7. (TIF) [file pone.0033730.s001.tif]

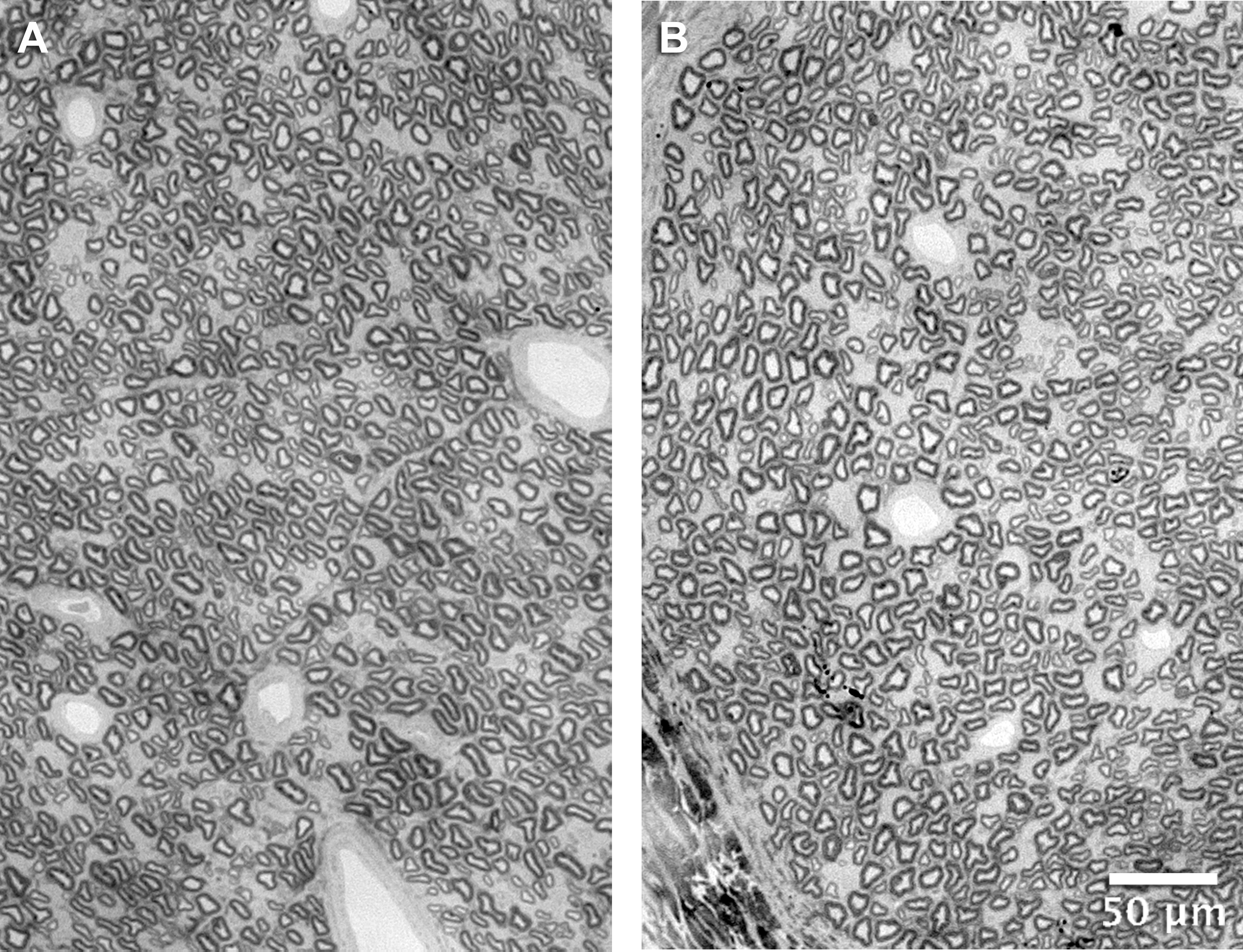

Supplement: Figure S2 — Analysis of peripheral nerve following 2 mg MIA injection. A, B - toluidine-blue stained semithin sections of common peroneal nerve ipsilateral (A) or contralateral (B) to 2 mg MIA injection at day 7 of the model. There is no visible axon degeneration, demyelination or inflammatory cell infiltrate (n = 4 per group). (TIF) [file pone.0033730.s002.tif]
