# Supplementary material for: Characterisation of a Peripheral Neuropathic Component of the Rat Monoiodoacetate Model of Osteoarthritis
Source: PLoS One. 2012 Mar 21;7(3):e33730. doi: 10.1371/journal.pone.0033730 (PMC3312347; doi:10.1371/journal.pone.0033730)
Supplement: Table S1 — Mean cartilage proteoglycan score for 2 mg MIA, 1 mg MIA and saline injected joints, as assessed using toluidine blue staining (See Fig. 1C, D .). Scoring reflects the degree of proteoglycan loss, with 12 corresponding to total loss over the entirety of both condylar surfaces, while 1 is undisrupted cartilage proteoglycan. n = 4×1 mg, 3×2 mg animals, +14d saline injected contralateral controls. (DOC) [file pone.0033730.s003.doc]

| Table 1: Cartilage proteoglycan score in MIA and saline injected knees | | |
| --- | --- | --- |
| MIA Dose group | Mean Proteoglycan Score / 12 | Standard error |
| 2mg | 6.3 | 1.20 |
| Saline injected contralateral control | 0.67 | 0.33 |
| 1mg | 5 | 1.78 |
| Saline injected 14d contralateral control | 1.5 | 0.65 |
