# Supplementary material for: Characterisation of a Peripheral Neuropathic Component of the Rat Monoiodoacetate Model of Osteoarthritis
Source: PLoS One. 2012 Mar 21;7(3):e33730. doi: 10.1371/journal.pone.0033730 (PMC3312347; doi:10.1371/journal.pone.0033730)
Supplement: Table S2 — 43 published studies which used the MIA model. Columns denote dose of monoiodoacetate used. The highest dose was 4.8 mg, the lowest was 0.01 mg. Studies in bold specifically assessed evoked pain responses at the hindpaw. Where multiple doses were used, a study is referenced at each dose. (DOC) [file pone.0033730.s004.doc]

**Table S2: 43 published studies which used the MIA model**

| **MIA Dose (mg)** |  |  |  |
| --- | --- | --- | --- |
| **< 1** | **1** | **2** | **3 or above** |
| Janusz, 2001 [1] | Guzman, 2003 [2] | **Fernihough, 2004 [3]** | **McDougall, 2006 [4]** |
| Brown, 2008 [5] | Pulichino, 2006 [6] | **Combe, 2004 [7]** | Shinkai, 2008a + b [8,9] |
| Chu, 2011 [10] | Ivanavicius, 2007 [11] | **Vermeirsch, 2007 [12]** | Schuelert, 2008 [13] |
| Guingamp, 1997 [14] | Piscaer, 2008 [15] | **Vonsy, 2009 [16]** | Chandran, 2009 [17] |
| Bove, 2003 [18] | Clements, 2009 [19] | Nemirovskiy, 2009 [20] | Honore, 2009 [21] |
| Pomonis, 2005 [22] | Baragi, 2009 [23] | **Rahman, 2009 [24]** | Boyce-Rustay, 2010 [25] |
| Cialdai, 2009 [26] | Cifuentes, 2010 [27] | Bar-Yehuda, 2009 [28] | Schuelert, 2010 [29] |
| Schuelert, 2009 [30] | Koh, 2010 [31] | **Kalff, 2010 [32]** | Swearingen, 2010 [33] |
| **Sagar, 2010 [34]** | Piscaer, 2011 [35] | Okamoto, 2010 [36] | **Schuelert, 2011 [37]** |
| Dumond, 2004 [38] | Bove, 2003 [18] | Ferreira‐Gomes, 2010 [39] | **Ferland, 2011 [40]** |
| Kobayashi, 2003 [41] | Pomonis, 2005 [22] | **Orita, 2011 [42]** | **Liu, 2011 [43]** |
|  | **Sagar, 2010 [34]** | **Ferreira-Gomes, 2008 [44]** | Bove, 2003 [18] |
|  | Cialdai, 2009 [26] | Cialdai, 2009 [26] | Pomonis, 2005 [22] |
|  |  |  | Schuelert, 2009 [30] |
|  |  |  | **Ferreira-Gomes, 2008 [44]** |
|  |  |  | **Sagar, 2010 [34]** |
|  |  |  | **Kobayashi, 2003 [41]** |
|  |  |  | Guingamp, 1997 [14] |
| 2 | 8 | 10 | 9 |

Supporting References

1. Janusz M, Hookfin E, Heitmeyer S, Woessner J, Freemont A, et al. (2001) Moderation of iodoacetate-induced experimental osteoarthritis in rats by matrix metalloproteinase inhibitors. Osteoarthritis and Cartilage 9: 751-760.

2. Guzman RE, Evans MG, Bove S, Morenko B, Kilgore K (2003) Mono-iodoacetate-induced histologic changes in subchondral bone and articular cartilage of rat femorotibial joints: an animal model of osteoarthritis. Toxicol Pathol 31: 619-624.

3. Fernihough J, Gentry C, Malcangio M, Fox A, Rediske J, et al. (2004) Pain related behaviour in two models of osteoarthritis in the rat knee. Pain 112: 83-93.

4. McDougall JJ, Watkins L, Li Z (2006) Vasoactive intestinal peptide (VIP) is a modulator of joint pain in a rat model of osteoarthritis. Pain 123: 98-105.

5. Brown KK, Heitmeyer SA, Hookfin EB, Hsieh L, Buchalova M, et al. (2008) P38 MAP kinase inhibitors as potential therapeutics for the treatment of joint degeneration and pain associated with osteoarthritis. Journal of inflammation (London, England) 5: 22.

6. Pulichino AM, Rowland S, Wu T, Clark P, Xu D, et al. (2006) Prostacyclin antagonism reduces pain and inflammation in rodent models of hyperalgesia and chronic arthritis. Journal of Pharmacology and Experimental Therapeutics 319: 1043.

7. Combe R, Bramwell S, Field MJ (2004) The monosodium iodoacetate model of osteoarthritis: a model of chronic nociceptive pain in rats? Neuroscience Letters 370: 236-240.

8. Shinkai N, Korenaga K, Mizu H, Yamauchi H (2008) Intra-articular penetration of ketoprofen and analgesic effects after topical patch application in rats. J Control Release 131: 107-112.

9. Shinkai N, Korenaga K, Takizawa H, Mizu H, Yamauchi H (2008) Percutaneous penetration of felbinac after application of transdermal patches: relationship with pharmacological effects in rats. J Pharm Pharmacol 60: 71-76.

10. Chu KL, Chandran P, Joshi SK, Jarvis MF, Kym PR, et al. (2011) TRPV1-related modulation of spinal neuronal activity and behavior in a rat model of osteoarthritic pain. Brain Res 1369: 158-166.

11. Ivanavicius SP, Ball AD, Heapy CG, Westwood FR, Murray F, et al. (2007) Structural pathology in a rodent model of osteoarthritis is associated with neuropathic pain: increased expression of ATF-3 and pharmacological characterisation. Pain 128: 272-282.

12. Vermeirsch H, Biermans R, Salmon PL, Meert TF (2007) Evaluation of pain behavior and bone destruction in two arthritic models in guinea pig and rat. Pharmacol Biochem Behav 87: 349-359.

13. Schuelert N, McDougall JJ (2008) Cannabinoid‐mediated antinociception is enhanced in rat osteoarthritic knees. Arthritis & Rheumatism 58: 145-153.

14. Guingamp C, Gegout-Pottie P, Philippe L, Terlain B, Netter P, et al. (1997) Mono-iodoacetate-induced experimental osteoarthritis. A dose-response study of loss of mobility, morphology, and biochemistry. Arthritis Rheum 40: 1670-1679.

15. Piscaer TM, Waarsing JH, Kops N, Pavljasevic P, Verhaar JAN, et al. (2008) In vivo imaging of cartilage degeneration using microCT-arthrography. Osteoarthr Cartil 16: 1011-1017.

16. Vonsy JL, Ghandehari J, Dickenson AH (2009) Differential analgesic effects of morphine and gabapentin on behavioural measures of pain and disability in a model of osteoarthritis pain in rats. European journal of pain (London, England) 13: 786-793.

17. Chandran P, Pai M, Blomme EA, Hsieh GC, Decker MW, et al. (2009) Pharmacological modulation of movement-evoked pain in a rat model of osteoarthritis. European Journal of Pharmacology 613: 39-45.

18. Bove SE, Calcaterra SL, Brooker RM, Huber CM, Guzman RE, et al. (2003) Weight bearing as a measure of disease progression and efficacy of anti-inflammatory compounds in a model of monosodium iodoacetate-induced osteoarthritis. Osteoarthr Cartil 11: 821-830.

19. Clements KM, Ball AD, Jones HB, Brinckmann S, Read SJ, et al. (2009) Cellular and histopathological changes in the infrapatellar fat pad in the monoiodoacetate model of osteoarthritis pain. Osteoarthr Cartil 17: 805-812.

20. Nemirovskiy OV, Radabaugh MR, Aggarwal P, Funckes-Shippy CL, Mnich SJ, et al. (2009) Plasma 3-nitrotyrosine is a biomarker in animal models of arthritis: Pharmacological dissection of iNOS' role in disease. Nitric Oxide 20: 150-156.

21. Honore P, Chandran P, Hernandez G, Gauvin DM, Mikusa JP, et al. (2009) Repeated dosing of ABT-102, a potent and selective TRPV1 antagonist, enhances TRPV1-mediated analgesic activity in rodents, but attenuates antagonist-induced hyperthermia. Pain 142: 27-35.

22. Pomonis JD, Boulet JM, Gottshall SL, Phillips S, Sellers R, et al. (2005) Development and pharmacological characterization of a rat model of osteoarthritis pain. Pain 114: 339-346.

23. Baragi VM, Becher G, Bendele AM, Biesinger R, Bluhm H, et al. (2009) A new class of potent matrix metalloproteinase 13 inhibitors for potential treatment of osteoarthritis: Evidence of histologic and clinical efficacy without musculoskeletal toxicity in rat models. Arthritis Rheum 60: 2008-2018.

24. Rahman W, Bauer C, Bannister K, Vonsy J, Dolphin A, et al. (2009) Descending serotonergic facilitation and the antinociceptive effects of pregabalin in a rat model of osteoarthritic pain. Molecular Pain 5: 45.

25. Boyce-Rustay JM, Simler GH, McGaraughty S, Chu KL, Wensink EJ, et al. (2010) Characterization of fasudil in preclinical models of pain. The Journal of Pain 11: 941-949.

26. Cialdai C, Giuliani S, Valenti C, Tramontana M, Maggi CA (2009) Effect of Intra-articular 4-(S)-amino-5-(4-{4-[2,4-dichloro-3-(2,4-dimethyl-8-quinolyloxymethyl)phenylsulfonamido]-tetrahydro-2H-4-pyranylcarbonyl} piperazino)-5-oxopentyl](trimethyl)ammonium chloride hydrochloride (MEN16132), a kinin B2 receptor antagonist, on nociceptive response in monosodium iodoacetate-induced experimental osteoarthritis in rats. Journal of Pharmacology and Experimental Therapeutics 331: 1025-1032.

27. Cifuentes D, Rocha L, Silva L, Brito A, Rueff-Barroso C, et al. (2010) Decrease in oxidative stress and histological changes induced by physical exercise calibrated in rats with osteoarthritis induced by monosodium iodoacetate. Osteoarthritis and Cartilage 18: 1088-1095.

28. Bar-Yehuda S, Rath-Wolfson L, Del Valle L, Ochaion A, Cohen S, et al. (2009) Induction of an antiinflammatory effect and prevention of cartilage damage in rat knee osteoarthritis by CF101 treatment. Arthritis Rheum 60: 3061-3071.

29. Schuelert N, Johnson MP, Oskins JL, Jassal K, Chambers MG, et al. (2010) Local application of the endocannabinoid hydrolysis inhibitor URB597 reduces nociception in spontaneous and chemically induced models of osteoarthritis. PAIN 152: 975-981.

30. Schuelert N, McDougall JJ (2009) Grading of monosodium iodoacetate-induced osteoarthritis reveals a concentration-dependent sensitization of nociceptors in the knee joint of the rat. Neuroscience Letters 465: 184-188.

31. Koh YH, Hong SH, Kang HS, Chung CY, Koo K-H, et al. (2010) The effects of bone turnover rate on subchondral trabecular bone structure and cartilage damage in the osteoarthritis rat model. Rheumatol Int 30: 1165-1171.

32. Kalff K, El Mouedden M, van Egmond… J (2010) Pre-treatment with capsaicin in a rat osteoarthritis model reduces the symptoms of pain and bone damage induced by monosodium iodoacetate. European Journal of Pharmacology 641: 108-113.

33. Swearingen CA, Chambers MG, Lin C, Marimuthu J, Rito CJ, et al. (2010) A short-term pharmacodynamic model for monitoring aggrecanase activity: injection of monosodium iodoacetate (MIA) in rats and assessment of aggrecan neoepitope release in synovial fluid using novel ELISAs. Osteoarthr Cartil 18: 1159-1166.

34. Sagar DR, Staniaszek LE, Okine BN, Woodhams S, Norris LM, et al. (2010) Tonic modulation of spinal hyperexcitability by the endocannabinoid receptor system in a rat model of osteoarthritis pain. Arthritis & Rheumatism 62: 3666-3676.

35. Piscaer TM, Müller C, Mindt TL, Lubberts E, Verhaar JAN, et al. (2011) Imaging of activated macrophages in experimental osteoarthritis using folate targeted animal SPECT/CT. Arthritis and rheumatism.

36. Okamoto M, Atsuta Y (2010) Cartilage degeneration is associated with augmented chemically-induced joint pain in rats: a pilot study. Clin Orthop Relat Res 468: 1423-1427.

37. Schuelert N, Johnson MP, Oskins JL, Jassal K, Chambers MG, et al. (2011) Local application of the endocannabinoid hydrolysis inhibitor URB597 reduces nociception in spontaneous and chemically induced models of osteoarthritis. Pain 152: 975-981.

38. Dumond H, Presle N, Pottie P, Pacquelet S, Terlain B, et al. (2004) Site specific changes in gene expression and cartilage metabolism during early experimental osteoarthritis. Osteoarthr Cartil 12: 284-295.

39. Ferreira‐Gomes J, Adães S, Sarkander J, Castro‐Lopes JM (2010) Phenotypic alterations of neurons that innervate osteoarthritic joints in rats. Arthritis & Rheumatism 62: 3677-3685.

40. Ferland CE, Laverty S, Beaudry F, Vachon P (2011) Gait analysis and pain response of two rodent models of osteoarthritis. Pharmacol Biochem Behav 97: 603-610.

41. Kobayashi K, Imaizumi R, Sumichika H, Tanaka H, Goda M, et al. (2003) Sodium iodoacetate-induced experimental osteoarthritis and associated pain model in rats. J Vet Med Sci 65: 1195-1199.

42. Orita S, Ishikawa T, Miyagi M, Ochiai N, Inoue G, et al. (2011) Pain-related sensory innervation in monoiodoacetate-induced osteoarthritis in rat knees that gradually develops neuronal injury in addition to inflammatory pain. BMC Musculoskelet Disord 12: 134.

43. Liu P, Okun A, Ren J, Guo R-C, Ossipov MH, et al. (2011) Ongoing pain in the MIA model of osteoarthritis. Neuroscience Letters 493: 72-75.

44. Ferreira-Gomes J, Adães S, Castro-Lopes JM (2008) Assessment of movement-evoked pain in osteoarthritis by the knee-bend and CatWalk tests: a clinically relevant study. The Journal of Pain 9: 945-954.
